# Incidence of Intraocular Lens Exchange after Cataract Surgery

**DOI:** 10.1038/s41598-019-49030-2

**Published:** 2019-09-09

**Authors:** Maram E. A. Abdalla Elsayed, Khabir Ahmad, Abdulelah A. Al-Abdullah, Rizwan Malik, Rajiv Khandekar, Hernan Martinez-Osorio, Marco Mura, Patrik Schatz

**Affiliations:** 1 Jeddah Eye Hospital, Jeddah, Saudi Arabia; 2 Research Department, King Khaled Eye Specialist Hospital, Riyadh, Saudi Arabia; 3 Vitreoretinal Division, King Khaled Eye Specialist Hospital, Riyadh, Saudi Arabia; 4 Glaucoma Division, King Khaled Eye Specialist Hospital, Riyadh, Saudi Arabia; 5 Anterior Segment Division, King Khaled Eye Specialist Hospital, Riyadh, Saudi Arabia; 6 Ophthalmology Department, University of Illinois, Chicago, USA; 7 Department of Ophthalmology, Clinical Sciences, Skane University Hospital, Lund University, Lund, Sweden

**Keywords:** Lens diseases, Outcomes research, Risk factors

## Abstract

Intraocular lens (IOL) exchange after cataract surgery is unusual but may be associated with suboptimal visual outcome. The incidence of IOL exchange has not been consistently estimated. Such information is invaluable when counseling patients prior to cataract surgery. We examined the incidence of, and indications and risk factors for, IOL exchange after cataract surgery. We also assessed visual outcome of eyes that had an IOL exchange. A cohort design was used to estimate the incidence of IOL exchange and a case-control design to identify factors associated with it. All phacoemulsification surgeries with IOL (n = 17415 eyes) during 2010–2017 and those that had a subsequent IOL removal or replacement during the same time period were identified (n = 34 eyes). The incidence of IOL exchange was 2 per 1000 surgeries (95% confidence interval [CI] 1 to 3) over 8 years. Eyes that underwent subsequent IOL removal or replacement were compared with eyes that had cataract surgery only (n = 47) across demographic and clinical characteristics. In a binary logistic regression analysis, two factors were significantly associated with IOL exchange/removal: an adverse event during cataract surgery (adjusted odds ratio [aOR] 19.45; 95% CI 4.89–77.30, P < 0.001) and a pre-existing ocular comorbidity (aOR 10.70; 95% CI 1.69–67.63, P = 0.021). The effect of gender was marginally significant (P = 0.077). Eyes that underwent IOL exchange or explantation were nearly two and a half times more likely to have a final best-corrected visual acuity of <20/60 compared to those that had cataract surgery alone (adjusted RR 2.60 95% CI, 1.13–6.02; P = 0.025).

## Introduction

Age-related cataract remains the leading cause of blindness worldwide, despite the fact that cataract surgery is one of the most frequently performed and cost effective surgical interventions to date^1–5^. The outcomes of cataract surgery are generally very good. However, potentially vision-threatening complications may occur in a small proportion of patients. Most of these complications (estimated incidence) have been extensively studied including endophthalmitis (0.05–0.15%)^6^, retinal detachment (0.39%)^7^, and expulsive hemorrhage (0.05–0.1%)^8^. Seemingly less severe and often seen as a late complication, the need for intraocular lens (IOL) exchange after primary cataract surgery is unusual and may lead to suboptimal visual outcome^9–15^. However the incidence of this surgery has not been consistently estimated previously. Here, we studied the incidence of IOL exchange among patients that had primary surgery at a single center over a period of 8 years. We also examined the risk factors for, and visual outcomes after intraocular lens (IOL) removal/exchange after cataract surgery.

## Results

### Incidence of IOL exchange

During 2010–2017, a total of 17415 eyes underwent phacoemulsification with IOL implantation in King Khaled Eye Specialist Hospital (KKESH). Of these, we initially identified 45 eyes of 45 patients who had IOL removal or exchange in the same period. Eleven eyes (of 11 patients) were excluded because these had phakic IOL removal with cataract surgery. Thus 34 eyes (of 34 patients) that underwent subsequent IOL removal or replacement during same period remained, equaling an overall cumulative incidence of IOL exchange/explantation of 2 per 1000 surgeries (95% confidence interval [CI] 1 to 3).

### Characteristics of eyes/individuals with IOL exchange/explantation and their controls

The demographic and clinical information for cases and controls is summarized in Table 1. Briefly, individuals/eyes that underwent IOL exchange/ex-plantation were almost comparable to controls in terms of most characteristics (i.e., age, pre-existing systemic comorbidities, and operated eye), except that eyes with IOL exchange had a greater mean axial length and a higher burden of pre-existing ocular comorbidities such as glaucoma (3 eyes), high myopia (2 eyes), and uveitis, pseudoexfoliation and Fuchs’ dystrophy and previous intraocular surgery (1 eye each) compared to control eyes.

**Table 1:** Characteristics of cases and controls.

Characteristic	IOL exchange/explantation group n = 34		Control group n = 47	
	Freq	%	Freq	%
Age (years)*	58.32 ± 13.51		58.26 ± 16.41	
Gender				
Male	20	58.8	22	46.8
Female	14	41.2	25	53.2
Eye				
Right	23	67.6	26	55.3
Left	11	32.4	21	44.7
Pre-existing systemic comorbidity^†^				
Yes	24	70.6	36	76.6
No	10	29.4	11	23.4
Pre-existing ocular comorbidity^‡^				
Yes	7	20.6	2	4.3
No	27	79.4	45	95.7
Axial length (millimeter)^*^	25.10 ± 2.14		24.10 ± 1.68	
Adverse event during cataract surgery^§^				
Yes	17	50	4	8.5
No	17	50	43	91.5
Type of surgery				
Phacoemulsification	20	58.8	43	91.5
Phaco + vitrectomy/other procedure	14	41.2	4	8.5
IOL position				
In the bag	25	73.5	44	93.6
Anterior chamber/sulcus	9	26.5	3	6.4

*Data are presented as means and SD.

^†^Frequent systemic comorbidities included diabetes and hypertension.

^‡^Pre-existing ocular comorbidities included glaucoma, high myopia, previous intraocular surgery, uveitis, pseudoexfoliation and Fuchs’ dystrophy.

^§^Frequent adverse events during cataract surgery included posterior capsular tear with or without vitreous prolapse, or subluxated intraocular lens.

### Factors associated with of IOL exchange/explantation

In the multivariable binary logistic regression analysis (Table 2), an adverse event during cataract surgery and a pre-existing ocular comorbidity were significantly associated with the outcome. The effect of gender was marginally significant and that of axial length was not statistically significant. Briefly, the risk of IOL exchange/explantation was higher among eyes with an eventful cataract surgery (adjusted odds ratio [aOR] 19.45; 95% CI 4.89–77.30, *P* < 0.001); among those with a pre-existing ocular comorbidity (aOR 10.70; 95% CI 1.69–67.63, *P* = 0.012), among males (aOR 2.94; 95% CI 0.89–9.73, P = 0.077), and among eyes with an axial length of ≥26 mm (aOR 2.92; 95% CI 0.75 11.28 *P* = 0.121), compared with their reference groups.

**Table 2:** Bivariate and multivariable binary logistic regression analysis of factors associated with IOL exchange/explantation.

Characteristic	Crude OR (95% CI)	*P*	Adjusted OR (95% CI)	*P*
Age, years				
≤59	1.28 (0.53–3.10)	0.586		
≥60	1.0			
Gender				
Male	1.62 (0.67–3.96)	0.287	2.94 (0.89–9.73)	0.077
Female	1.0			
Eye				
Right	1.69 (0.67–4.24)	0.264		
Left	1.0			
Pre-existing systemic comorbidity*				
Yes	0.73 (0.27–1.99)	0.543		
No	1.0			
Pre-existing ocular comorbidity^†^				
Yes	5.83 (1.13–30.14)	0.035	10.70 (1.69–67.63)	0.012
No	1.0			
Axial length, mm				
≤25	1.0			
≥26	2.38 (0.80–7.09)	0.150	2.92 (0.75–11.28)	0.121
Adverse event during cataract surgery^‡^				
Yes	10.75 (3.16–36.61)	<0.001	19.45 (4.89–77.30)	<0.001
No	1.0		1.0	
Type of surgery				
Phacoemulsification	1.0			
Phaco + vitrectomy/other procedure	7.53 (2.20–25.78)	0.001		
IOL position				
In the Bag	1.0			
Anterior chamber/sulcus	5.28 (1.31–21.32)	0.019		

^*^Frequent systemic comorbidities included diabetes and hypertension.

^†^Pre-existing ocular comorbidities included glaucoma, high myopia, previous intraocular surgery uveitis, pseudoexfoliation and Fuchs’ dystrophy.

^‡^Frequent adverse events during cataract surgery included posterior capsular tear with or without vitreous prolapse, or subluxated intraocular lens.

### Indication for IOL exchange and location of re-implanted IOL

The most frequent indications for IOL exchange (Table 3) were: In-the-bag subluxation in 8 eyes (23.5%), out-of-the bag subluxation and refractive error in 7 each (20.6% each) and uveitis in 6 (17.6%). Of 7 eyes with refractive error, 4 eyes had undergone a previous myopic LASIK (laser *in situ* keratomileusis) procedure. Of the 3 remaining eyes, one had preoperative axial length measure using ultrasound instead of optical biometry, because of mature cataract. The remaining 2 eyes had received the wrong IOL power without any clear reason. For all the 7 eyes that were given wrong IOL power, the final IOL position (after IOL exchange) was in the bag.

**Table 3:** Indication for IOL exchange and location of re-implanted IOL after cataract surgery (n = 34 eyes of 34 patients).

Characteristic	Number	%
*Indication*		
In the bag subluxation	8	23.5
Out of the bag subluxation	7	20.6
Refractive error	7	20.6
Uveitis	6	17.6
Opacification of intraocular lens	3	8.8
Corneal decompensation	2	5.9
Trauma	1	2.9
*Location of re-implanted IOL*		
Sulcus	9	26.5
In the bag	8	23.5
Iris fixated	5	14.7
Scleral fixated	5	14.7
Anterior chamber	4	11.8
Aphakia	3	8.8

Of the explanted IOLs, 25 were one-piece acrylic hydrophobic, 2 were three-piece acrylic hydrophobic and 7 were one-piece polymethyl methacrylate. The most frequent locations of the re-implanted IOLs were: sulcus in 9 eyes (26.5%), in-the-bag in 8 (23.5%), iris fixated and scleral fixated in 5 each (14.7% each) and anterior chamber in 4 (11.8%).

Anterior vitrectomy and pars plana vitrectomy (PPV) were needed at the time of IOL exchange in 5 and 6 eyes, respectively, where PPV alone was done for those that had posterior IOL dislocation. Another 2 eyes needed subsequent PPV for reasons unrelated to the IOL exchange; 1 of these for diabetes tractional detachment and vitreous hemorrhage and 1 for panuveitis.

### Visual outcome of IOL exchange/explantation

A higher proportion of eyes that underwent IOL exchange or explantation had suboptimal vision (best corrected visual acuity [BCVA] <20/60) than the control eyes at the most recent visit (Table 4). Eyes that underwent IOL exchange or explantation were three times (crude relative risk [RR] 3.11 [95% CI, 1.35–7.15]; *P* = 0.008) more likely to have a BCVA of <20/60 than those that had cataract surgery alone. This relative risk remained high and statistically significant even after adjustment for baseline BCVA (adjusted RR 2.60 [95% CI, 1.13–6.02]; *P* = 0.025).

**Table 4:** Best corrected visual acuity at most recent follow up in eyes that had an IOL exchange/explantation after cataract surgery, and in control eyes that had cataract surgery only.

	Best corrected visual acuity at the last follow up		Relative risk (95 CI %) for BCVA <20/60*	
Group**	20/20–20/60	<20/60–20/200		
	Freq (%)	Freq (%)	Crude	Adjusted
IOL exchange/explantation(n = 34 eyes)	16 (47.1)	18 (52.9)	3.11 (1.35–7.15)	2.60 (1.13–6.02)
Control (n = 47 eyes)	39(83.0)	8 (17.0)	1.0	1.0

^*^Adjusted for baseline BCVA using Cox’s regression. P values for crude and adjusted RR = 0.008 and 0.025.

**Of the 34 eyes in the IOL exchange/explantation group, 11(32.4%) and 7(20.6%) had a BCVA <20/60–20/200 and <20/200, respectively. By contrast, of 47 eyes in the control group, 6(12.8%) and 2 (4.3%) had a BCVA <20/60–20/200 and <20/200, respectively.

## Discussion

Several retrospective studies have looked at the risk factors for and visual outcomes after IOL removal/exchange^10–15^. However, to the best of our knowledge this is the first study to report the incidence of IOL removal/exchange (2 per 1000 cataract surgeries (95% confidence interval [CI] 1 to 3, over 8 years, corresponding to an average annual incidence of 2.5 per 10,000). This information is invaluable for counseling patients prior to cataract surgery about the risk of an IOL exchange and its sequelae.

IOL removal/exchange incidence estimates that exist in the literature are all based on one specific subgroup of these patients; namely those that had late IOL dislocations^16–20^. Furthermore, most of the patients examined in those studies only needed IOL repositioning as opposed the exchange or replacement^17–20^. Focusing on late IOL dislocation, Clark *et al*. reported the 5-year cumulative incidence of IOL dislocation to be 0.3% in a population-based study, with an increase in the rate of this complication during a study period of 22 years^16^. This figure is somewhat higher compared to our study (0.2% over 8 years). However, it is notable that Clark *et al*. did not mention the degree of dislocation and whether IOL exchange was required or not^16^.

Jakobsson *et al*. characterized the risk factors of late IOL dislocation in a sample of 84 eyes, finding an overrepresentation of pseudoexfoliation (PXF), glaucoma and cataract surgeries complicated by zonular dehiscence^17^. They used data from the Swedish National Cataract Register between 2004 and 2006 to estimate the incidence of IOL repositioning surgery, by dividing the number of IOL repositioning surgeries by the total number of pseudophakic individuals during the same period, arriving at an annual incidence of 0.05%, which is slightly higher than the average annual incidence described in our study (0.025%)^17^. However only 7% of their patients required IOL exchange or removal and the remaining patients had scleral suturing of the dislocated IOL^17^. Monestam *et al*. followed 810 patients for 10 years after cataract surgery and identified 5 patients that required further surgery for dislocated IOL, resulting in a 10 year incidence of 0.6% for this complication^18^. Kinga Dabrowska-Kloda *et al*. estimated the annual incidence of late in-the-bag IOL dislocation over a 21-year period, reviewing 123 patients (eyes) and an equal number of controls, and data from the Swedish National Cataract Registry and life tables from official statistics on the Swedish population^19^. The annual incidence varied between 0 and 0.08%. The cumulative risk was 0.09% and 0.55% over 5 and 10 years, respectively. Risk factors included PXF, zonular dehiscence, pseudophakodonesis and increased axial length^19^.

These studies have reported slightly higher incidence estimates compared to our study, which may be related to the fact that most patients in these studies needed only IOL repositioning^17–20^ as opposed to IOL exchange, whereas our study focused specifically on IOL removal/exchange. On the other hand such differences may, in part, be due to differences in surgical approach (which is partly determined by surgeon’s preference and which we could not control for in our retrospective study) to manage eyes with IOL dislocations.

Jin *et al*. 2007 focused on IOL exchange due to postoperative refractive error^20^. Incorrect corneal power estimation, followed by error in measurement of axial length and inserting a wrong IOL were the most common reasons for IOL exchange^20^. In this study, we identified 7 eyes that needed IOL exchange due to refractive error (Table 3). Of these, 4 had incorrect corneal power estimation related to previous myopic LASIK procedures, leading to a low postoperative corneal power. This, in turn, tends to lead to an underestimation of the effective lens position (ELP) and therefore to an underestimation of the correct IOL power for emmetropia. Of the 3 remaining eyes, one had preoperative axial length measure using ultrasound instead of optical biometry, because of mature cataract. Ultrasound tends to lead to an underestimation of the correct axial length and subsequent overestimation of the correct IOL power, due to compression of the globe during the ultrasound examination.

The indications for IOL removal/exchange have been examined in several previous studies. Davies *et al*. found that the indications IOL exchange were IOL dislocation to the posterior pole, dissatisfaction with multifocal IOL and uveitis^9^. In a study by Chan *et al*., the most important indication for in the bag IOL exchange were IOL decentration, caused by complicated surgeries, and high myopia^11^. The most common indication for sulcus IOL exchange was glaucoma-hyphema-uveitis syndrome, especially for the one-piece IOL^11^. Jones *et al*. found that IOL dislocation, refractive error, patient dissatisfaction and implant opacification were the main indications for IOL exchange^10^. Neuhann *et al*. presented results of 105 eyes from 100 patients undergoing IOL exchange/explant. In their study, the main indication was subluxation with IOL dislocation, especially associated with pseudoexfoliation syndrome^12^. Incorrect IOL power calculation, calcification and corneal endothelial decompensation were also found^12^. In our study, the most common indication for IOL exchange/removal was also IOL dislocation (Table 3).

Jakobsson *et al*. 2013 examined the outcomes after surgical repositioning of IOLs after late dislocation and found that 94% could be repositioned using scleral sutures. Pseudoexfoliation was detected in 57% of the eyes, which was also reflected in the present study, where ocular comorbidity including pseudexfoliation, was an independent risk factor for IOL exchange (Table 2). They found that 13 eyes (out of 91) had additional surgical procedures, and 3 of those had postoperative retinal detachments. More than half of the eyes obtained visual acuities more than 20/40 and 23% of the eyes had a decrease in the visual acuity following the surgery^21^. In our study, eyes that underwent IOL exchange were nearly two and a half times more likely to have a final visual acuity of <20/60 compared to the control eyes (Table 4).

Kamiya *et al*. found that the most common complaints for multifocal IOL removal were waxy vision, glare and halos, blurred vision (at far, at near, or at intermediate distances), and dysphotopsia,. The most common reasons for multifocal IOL removal were decreased contrast sensitivity, followed by photic phenomenon, unknown origin including neuroadaptation failure, incorrect IOL power, preoperative excessive expectation, IOL dislocation/decentration, and anisometropia. The mean ± SD axial length was 25.13 ± 1.83 mm in their study^22^. Myopia was also overrepresented in the IOL removal/exchange group in our study, and 4 of the patients had undergone previous LASIK for high myopia (>6D), leading to difficulties in estimating the central corneal power. However, in our final multivariable model, axial length, when used as a categorical variable, did not turn out to be significantly associated with IOL exchange, although it was significant when used as a continuous variable (data not shown). This is possibly because of a relatively limited sample size in our study (Table 2).

In our study, the presence of an intraoperative adverse event and a pre-existing ocular co-morbidity (such as pseudexfoliations) were significant predictors of IOL exchange. This finding generally concur with existing literature and is plausible.

Limitations of this study include its retrospective design and the inability to control or adjust for variability regarding surgeons preferred technique of IOL exchange or choice of IOL exchange versus IOL reposition. The figure of two IOL exchanges/removals per 1000 cataract surgeries may be an underestimate given that the follow-up time after cataract surgery was at most 8 years in our study. For the calculation of cumulative incidence of an event, the specified time interval should generally be the same for all members of a group. However, it may vary in certain situations^23^. A sufficiently large multi-center prospective study is warranted to more accurately estimate the true incidence of IOL exchange/removal and other rare complications of cataract surgery and to examine their risk factors and visual outcomes.

We conclude that IOL exchange is rare yet may affect the final visual outcome adversely. The most frequent indication was IOL dislocation. Two factors were significantly associated with IOL exchange/explantation: an adverse event during cataract surgery, and a pre-existing ocular comorbidity. Eyes that underwent IOL exchange or explantation were nearly two and a half time times more likely to have a final visual acuity of <20/60 compared to those that had cataract surgery alone.

## Methods

This retrospective study was undertaken at the King Khaled Eye Specialist Hospital, Riyadh, a tertiary eye care hospital. Ethics approval was obtained from the Institutional Review Board of KKESH. Informed consent for intraocular surgery was obtained from all patients. The research followed the tenets of the Declaration of Helsinki. A cohort design was employed to estimate the incidence of IOL exchange and a case-control design was used to identify factors associated with it.

The main outcome was the cumulative incidence of IOL exchange which was defined as the number of eyes that underwent IOL exchange/explantation, divided by the total number of eyes that underwent cataract surgery with IOL during the study period^23^. To achieve this objective, all eyes that underwent phacoemulsification cataract surgery with IOL implantation during 2010–2017 and had a subsequent IOL removal or replacement during the same time period were included in the study. Eyes that underwent phakic IOL removal with phacoemulsification and IOL were excluded. Diagnostic coding was used to identify the total number of cataract surgeries with IOL and those that had a subsequent exchange/explantation.

For the risk-factor component of the study, all eyes that underwent IOL exchange (n = 34) were compared with a subset of eyes that had cataract surgery alone (n = 47) during the same period. Specifically, a control eye/patient for a given case eye/patient was selected from the complete list of entries coded as “phacoemulsification with IOL”, where the first entry fulfilling the criterion of age matching the patients age ±5 years, was selected as a control.

A structured proforma (Supplemental File 1) was used to collect data from medical records on age, gender, pre-operative characteristics, indication for IOL exchange/explantation, the type of IOL that was removed, operative and postoperative complications, and visual outcomes.

All case and control eyes had baseline preoperative IOL calculations using Zeiss IOL Master 500 (Carl Zeiss Meditec, AG Jena, Germany), except where hazy media (such as mature cataract) precluded the use of optical biometry. In such cases, applanation ultrasound biometry (Cinescan S or Aviso, Quantel Medical Cedex - France) was used to estimate the axial length. The Holladay 2 formula was routinely used as the standard IOL calculation formula. However other formulas could be requested in addition, at the discretion of the surgeon. Emmetropia or mild myopia was the refractive target for all patients and controls. Visual acuity was measured in Snellen format.

### Statistical analysis

All data were entered in a Microsoft Excel (Microsoft Corporation, Redmond, WA, USA) database by a trained data entry operator and analyzed using SPSS for Windows version 20.0 (IBM SPSS, Inc., Chicago, IL, USA). For the present analysis, pre-existing ocular comorbities were grouped into binary categories with “Yes” indicating the presence of the condition and “No” otherwise, as were the pre-existing systemic comorbidities, adverse event during cataract surgery, type of surgery, and IOL position. Age was categorized as ≤59 years and ≥60 years, and axial length as ≤25 mm and ≥26 mm. Counts and percentages were used to describe categorical data and means (with SD) were computed to describe continuous variables.

A binary logistic regression analysis was performed to identify factors associated with IOL exchange/explantation. Odds ratios (OR) with 95% confidence intervals (CI) were calculated to indicate an association. Nine variables were examined in the univariate analysis. Of these, adverse event during cataract surgery, pre-existing ocular comorbidity, axial length, type of surgery and IOL position were significantly associated with IOL exchange/explantation. Both type of surgery and IOL position were not considered for the multivariable logistic regression models as they were strongly associated with an adverse event during cataract, as indicated by a high phi coefficients of 0.63 and 0.71, respectively. The final model included adverse event during cataract surgery, pre-existing ocular comorbidity, and axial length was adjusted for gender due to slight gender imbalance between case and control eyes.

Next, the best-corrected visual acuity (BCVA) at the last follow up was compared between eyes with and without subsequent IOL exchange/explantation. Using cox regression analysis, relative risk (RR) with 95% CIs were calculated to determine the association between IOL exchange/explantation and BCVA <20/60 at the last follow up. For all the analyses, 2-sided p-values were calculated. A p-value < 0.05 was considered significant. Supplemental File 1: data collection form and Supplemental File 2: Excel datasheet are appended.

## Supplementary information

Supplementary information 1

Supplementary information 2
