# Supplementary material for: Incidence of Intraocular Lens Exchange after Cataract Surgery
Source: Sci Rep. 2019 Sep 9;9:12877. doi: 10.1038/s41598-019-49030-2 (PMC6733834; doi:10.1038/s41598-019-49030-2)
Supplement: Supplementary file 1 — Supplementary information 1 [file 41598_2019_49030_MOESM1_ESM.docx]

**Incidence of Intraocular Lens Exchange after Cataract Surgery.**

***Short title****:* Incidence of intraocular lens exchange.

**Authors:** Maram EA Abdalla Elsayed MD, Khabir Ahmad MD PhD, Abdulelah A Al-Abdullah MD, Rizwan Malik MD PhD, Rajiv Khandekar MD, Hernan Martinez-Osorio, Marco Mura MD, Patrik Schatz MD PhD.

**Data collection form**

**(A) Demographic information:**

- **MRN: ____________**
- **DOB: YYYY**
- **Gender: 1=M/ 2=F**

**(B) Information of eye before 1^st^ cataract surgery with IOL**

- Eye : 1=OD/ 2=OS
- BCVA: ________
- Axial length: ______ mm
- IOP: ___ mmHg
- Systemic comorbidity: 1=Y/ 2=N Specify: _________

**Ocular status/history before primary surgery**

- Fuchs dystrophy 1=Y/ 2=N
- *Pseudoexfoliation*: 1=Y/ 2=N
- *Glaucoma*: 1=Y/ 2=N
- Retinitis pigmentosa 1=Y/ 2=N
- PDR 1=Y/ 2=N
- High myopia (≥ 6.0D): 1=Y/ 2=N
- Uveitis 1=Y/ 2=N
- Evidence of *ocular Trauma*: 1=Y/ 2=N
- *Previous intraocular surgery*: 1=Y/ 2=N If yes, type of surgery:

**(C) Information on Cataract surgery with IOL**

**Date of surgery**: ________ (DD/MM/YYYY)

**Group:**

**1= Patient**

**2= Control**

**Type of surgery**:

- 1= Phaco
- 2= Phaco + ant vitrectomy
- 3= Phaco +post vitr (+/- ant vitr)

**IOL position**:

- 1= In the bag
- 2= Sulcus
- 3= Anterior chamber
- 4= Iris fixated
- 5= Scleral fixated

**CTR used** 1=Y/ 2=N

**Intraoperative adverse event noted/managed**: 1=Y/ 2=N

If Yes:

- 1= Posterior capsular tear
- 2= Vitreous prolapse
- 3= Iris prolapse
- 4= Endothelial touch
- 5= Subluxated IOL
- 6= Other, specify:

**(D) Information regarding time between IOL implant and exchange:**

**OCULAR SURGERY performed in period between primary and secondary surgery (for controls: until last follow up).**

**Surgery performed**: 1=Y/ 2=N

**If Yes:**

- 1= Pars plana vitrectomy
- 2= Iol reposition
- 3= Trabeculectomy
- 4= Trabeculoplasty
- 5= Retinal laser
- 6= Refractive
- 7= Other, specify:

**YAG done during this interval?**

- YAG capsulotomy 1=Y/ 2=N If yes, date: DD/MM/YYYY

**Ongoing glaucoma medications**: 1=Y/ 2=N

**Medications for ocular inflammation** 1=Y/ 2=N

**(E) Details of IOL explant:**

Date of exchange: DD/MM/YYYY

Type of IOL re-implant:

- 1= Iris fixated
- 2= Anterior chamber
- 3= Sulcus
- 4= In the bag
- 5= Aphakia

Indication:

- 1= High residual RE
- 2= Uveitis
- 3= Iol opacification
- 4= Corneal decompensation
- 5= Cracked or opacified or multifocal IOL with dissatisfied patient
- 6= Chronic uveitis
- 7= Recurrent hyphema
- 8= Trauma
- 9= In the bag Subluxation
- 10= Out of the bag Subluxation

**Type of IOL removed:**

- 1= One-piece acrylic (hydrophilic)
- 2= One-piece acrylic (hydrophobic)
- 3= Three-piece acrylic (hydrophobic)
- 4= One piece PMMA.
- 5= Multifocal=4

**Type of IOL used during the exchange surgery:**

- 1= One-piece acrylic (hydrophilic)
- 2= One-piece acrylic (hydrophobic)
- 3= Three-piece acrylic (hydrophobic)
- 4= One piece PMMA
- 5= Multifocal
- 6= Aphakia

**Intraoperative complications:**

- 1= Posterior capsular rupture
- 2= Zonular dehiscence

**Postoperative complications:**

- 1= Cystoid macular oedema
- 2= Glaucoma
- 3= Hypotony
- 4= Corneal decompensation
- 5= Recurrent IOL subluxation/luxation
- 6= Vitreous hemorrhage or Retinal detachment
- 7= Choroidal hemorrhage
- 8= Hyphema
- 9= Uveitis

**(F) Last follow-up:**

Date of last F/U: DD/MM/YYYY

BCVA:

IOP: _____ mmHg

Glaucoma medications: 1=Y/2=N:

Location of IOL:

- 1= Iris fixated
- 2= Anterior chamber
- 3= Sulcus
- 4= In the bag
- 5= Aphakia

Ocular inflammation if any: 1=Y/2=N

Medication ongoing: 1=Y/2=N: Type of medication:
